# Does anxiety explain why math-anxious people underperform in math?

**DOI:** 10.1038/s41539-023-00156-z

**Published:** 2023-03-21

**Authors:** Richard J. Daker, Sylvia U. Gattas, Elizabeth A. Necka, Adam E. Green, Ian M. Lyons

**Affiliations:** 1 Department of Psychology, Georgetown University, Washington, D.C. USA; 2 Department of Experimental Psychology, University of Oxford, Oxford, UK; 3 National Institutes on Aging, National Institutes of Health, Bethesda, MD USA

**Keywords:** Human behaviour, Human behaviour

## Abstract

Math-anxious people consistently underperform in math. The most widely accepted explanation for *why* this underperformance occurs is that math-anxious people experience heightened anxiety when faced with math, and this in-the-moment anxiety interferes with performance. Surprisingly, this explanation has not been tested directly. Here, using both self-report and physiological indices of anxiety, we directly test how much in-the-moment anxiety explains math-anxious underperformance. Results indicate that in-the-moment anxiety indeed explains why math-anxious people underperform—but only partially, suggesting a need to seriously consider alternative mechanisms. Results also showed that while some highly math-anxious individuals—those with high levels of heart rate variability—experienced less in-the-moment anxiety, they nevertheless performed no better at math. For these individuals, math-anxious underperformance must occur for reasons unrelated to in-the-moment anxiety. More broadly, our findings point to substantial individual heterogeneity in the mechanisms underlying math-anxious underperformance. Accounting for this mechanistic heterogeneity may prove vital for optimally boosting math performance in math-anxious individuals.

## Introduction

While we tend to think that what determines how well we perform in key situations comes down to our level of ability or our preparedness, a great deal of work has demonstrated the pernicious effects that negative emotions, and in particular feelings of anxiety, can have on our cognitive performance^1–3^. One of the most well-studied examples of the negative effects that feelings of anxiety can have on cognitive performance is the consistent finding that those higher in math anxiety (i.e., feelings of anxiety specific to math) underperform in math compared to their less math-anxious peers^4–8^. This math-anxiety-related underperformance is not limited to lab-based math tasks—math-anxious students (those with high scores on trait-level math anxiety questionnaires) consistently underperform in classes that involve math, even after accounting for general anxiety^4,9^. This underperformance can have important consequences: High-paying jobs in STEM that rely on math often go unfilled^10^, and math skills are consistently predictive of important life outcomes—including financial and even health outcomes^11–13^. It is crucial, therefore, that we understand *why* math anxiety is associated with underperformance so that we can optimally intervene to help all students fulfill their potential in math.

So why is it that math-anxious people underperform in math? The most prominent explanation for why trait-level math anxiety predicts poor math performance is that, when faced with math, people who are high in trait-level math anxiety experience heightened in-the-moment anxiety (i.e., state anxiety), and it is this heightened in-the-moment anxiety that interferes with performance^14,15^. This is thought to occur because state anxiety co-opts working memory resources that are vital for successfully completing demanding math problems^2,16,17^. However, while several studies have provided evidence suggesting that reduced working memory and goal-directed processing resources play a role in math-anxious underperformance^16,18–26^, we know of no work that has directly tested whether or how much feelings of in-the-moment anxiety explain why math-anxious people underperform in math. Some past work has assessed interrelations among trait-level math anxiety, state-level math anxiety, and math achievement^27,28^ (for similar work in the statistics anxiety literature, see Macher et al.^29^), but no work to our knowledge has assessed how much in-the-moment anxiety can explain why math-anxious people underperform. This is a critical gap in our understanding—if indeed math-related state anxiety plays a primary role in explaining why math-anxious people underperform in math, then future intervention efforts to boost math performance should largely be devoted to finding ways to reduce this in-the-moment anxiety^30,31^. On the other hand, if empirical work demonstrated that in-the-moment anxiety does *not* play a large role in explaining why people high in trait-level math anxiety underperform in math, then future intervention research would be better aimed elsewhere.

When investigating how much in-the-moment anxiety explains why math-anxious people underperform, we also found it crucial to consider the role that emotion regulation processes might play. Emotion regulation refers to a diverse array of processes by which the individual influences the type, intensity, duration, and expression of emotions^32,33^. Importantly, many existing math anxiety interventions are based on promoting emotion regulation strategies^30,31,34–36^, with the rationale that providing people with effective emotion regulation tools may allow math-anxious people to better manage their feelings of in-the-moment anxiety and therefore perform better. This rationale is well-supported in the emotion regulation literature, as several studies have provided evidence that emotion regulation techniques can boost test performance^2,37–41^. However, past mechanistic work on math anxiety has by and large ignored the emotion regulation tools that individuals bring to the table themselves (e.g., absent interventions intended to increase emotion regulation abilities; see Daches Cohen, Korem, & Rubinsten^42^ for an example of this kind of work). There is substantial evidence that people vary in their ability to regulate negative emotions^43,44^. These trait-level differences in emotion regulation translate to state-level differences in how much anxiety individuals experience in anxiety-inducing situations: those with strong emotion regulation skills are able to better downregulate anxiety in the face of stressors compared to those with weak emotion regulation skills^44^. In the case of math anxiety, it seems quite plausible that math-anxious people with high levels of trait emotion regulation may be able to remain calm in the face of math. Conversely, math-anxious people with poor emotion regulation may become particularly anxious when they have to do math. If it were indeed the case that differences in emotion regulation in part determined just how anxious math-anxious people get when faced with math, it could very well mean that differences in emotion regulation also play an important role in determining exactly how much math-anxious people underperform—those with worse emotion regulation may underperform more than those with better emotion regulation.

To address these questions, we collected standard self-report measures of trait-level math anxiety several days prior to bringing participants into the lab. Once in the lab, we employed an experimental paradigm in which participants anticipated and eventually completed several blocks of a difficult mental math task (similar to the paradigm used in Lyons & Beilock^45,46^). To enable inferences that are specific to math, the same was done with a difficult word task.

To ask whether in-the-moment anxiety could explain math-anxious underperformance, we had to determine a way to index in-the-moment anxiety associated with math. Thus, we collected multiple indices of state anxiety while participants anticipated, completed, and just after completing each of the math and word tasks. These included self-report measures of ‘state’ anxiety^47,48^ in addition to multiple physiological indices often linked to anxiety: skin conductance, heart rate^49^, and a highly specific measure of sympathetic responsivity (pre-ejection period, PEP^50,51^). The vast majority of past work on math anxiety has only included trait-level measures^5^, with only a relatively small amount of studies including state-level measures. As a result, “math anxiety” is conceptualized as a trait-level measure in the vast majority of the literature, a convention we also adopt in this work. The past work that has assessed associations between trait- and state-level math anxiety has demonstrated that trait-level math anxiety is often associated with self-report measures of state anxiety associated with math^47,52^, but only inconsistent associations with physiological measures have been observed^49^. Using a combination of self-report measures as well as various physiological ones ensured the highest odds that, if indeed indices of in-the-moment anxiety explained math-anxious underperformance, we would detect it.

We faced the same measurement question in quantifying the emotion regulation skills participants brought to the table. We used a self-report measure (the Emotion Regulation Questionnaire), which is predictive of negative emotional responding to stressful situations^43,44^. In addition, we collected an objective physiological measure of high-frequency heart rate variability (HF-HRV), which reflects the degree to which a person’s resting heart rate is under parasympathetic control^53^. While the end-organ for this index is the heart, resting HF-HRV is also thought to index the effectiveness of central nervous system emotion regulation processes^54–56^. Evidence for this comes both from work showing that HF-HRV is strongly associated with functional connectivity in neural circuits that support emotion regulation (e.g., between prefrontal cognitive control regions and subcortical regions including the amygdala^57,58^) and from work showing that individuals with higher HF-HRV cope better with stress^56,59,60^.

In sum, in the present work, we addressed critical gaps in our understanding of math anxiety by directly testing (1) how much in-the-moment anxiety associated with math can explain why math-anxious people underperform and (2) whether these dynamics play out differently as a function of differences in emotion regulation. In addressing these questions, we employed both self-report and physiological indices of in-the-moment anxiety (i.e., state anxiety) and emotion regulation so that we could also address which type of measures (self-report or physiological) hold greater utility in explaining associations between math anxiety and math performance.

## Results

Participants completed online questionnaires including measures of math anxiety, general trait anxiety, and emotion regulation habits followed by an in-lab experimental session. In the experimental session, we first collected a 3-min passive baseline measure of all physiology measures (heart rate, skin conductance levels, pre-ejection period, and high-frequency heart rate variability). After this, the main experiment began. In each of eight blocks of the experimental paradigm, participants anticipated and eventually completed either a difficult math task or a difficult word task. This design allowed us to glean participants’ own reports of how anxious they were feeling before and after completing math as well as their physiological responses to both anticipation and completion of math. For descriptive statistics, see Table 1. For a correlation matrix showing associations between all variables, see Supplementary Table 1. All statistical tests reported are two-sided.

**Table 1:** Descriptive Statistics.

Measure	N	Mean (SE)	Min	Max	Range
Math Anxiety	83	31.13 (2.01)	1	80	79
General Trait Anxiety	83	45.13 (1.04)	24	70	46
ERQ Reappraisal	83	29.70 (0.67)	17	42	25
ERQ Suppression	83	13.98 (0.56)	4	27	23
Math Performance	83	0.74 (0.02)	0.18	1.00	0.82
Word Performance	83	0.83 (0.02)	0.41	0.99	0.58
Anticipatory Self-Reported State Anxiety - Math	83	2.84 (0.13)	1.00	6.27	5.27
Anticipatory Self-Reported State Anxiety - Word	83	2.54 (0.12)	1.00	5.40	4.40
Post-Task Self-Reported State Anxiety - Math	83	2.91 (0.14)	1.00	7.00	6.00
Post-Task Self-Reported State Anxiety - Word	83	2.36 (0.14)	1.00	6.25	5.25
Task SCL - Math	80	7.63 (0.72)	0.08	25.88	25.80
Task SCL - Word	80	7.23 (0.69)	0.08	27.02	26.95
Task HR - Math	78	74.08 (0.96)	53.05	96.63	43.57
Task HR - Word	78	72.93 (1.01)	50.15	98.89	48.74
Task PEP - Math	78	102.94 (1.62)	69.88	133.00	63.12
Task PEP - Word	77	102.97 (1.74)	55.12	131.62	76.50
Task HF-HRV - Math	78	6.58 (0.10)	4.30	8.57	4.26
Task HF-HRV - Word	78	6.81 (0.11)	4.56	9.16	4.60
Anticipatory SCL - Math	77	8.14 (0.76)	0.08	26.70	26.62
Anticipatory SCL - Word	79	7.97 (0.74)	0.08	28.23	28.15
Anticipatory HR - Math	75	73.74 (1.02)	52.43	101.21	48.78
Anticipatory HR - Word	77	73.68 (1.02)	50.94	100.14	49.20
Anticipatory PEP - Math	75	103.60 (1.58)	74.75	132.50	57.75
Anticipatory PEP - Word	76	103.10 (1.64)	62.50	132.12	69.62
Anticipatory HF-HRV - Math	75	6.32 (0.10)	3.99	8.40	4.41
Anticipatory HF-HRV - Word	77	6.43 (0.10)	4.20	8.67	4.47
Baseline SCL	83	5.25 (0.62)	0.06	30.19	30.13
Baseline HR	79	75.72 (1.11)	50.37	104.58	54.22
Baseline PEP	79	94.47 (1.61)	48.50	123.50	75.00
Baseline HF-HRV	79	6.15 (0.13)	2.67	8.62	5.95

Descriptive statistics for all variables are shown.

To begin our analyses, we first asked whether we saw evidence of math-anxious underperformance in this sample. We observed a significant negative correlation between trait-level math anxiety and math performance (*r*(81) = −0.469, *p* = 8E−6), showing that, consistent with decades of past research^4–8^, math-anxious people underperformed in math. Moreover, this association held when controlling for general anxiety and word performance (*r*_partial_(79) = −0.432, *p* = 6E−5), ruling out either general anxiety or global anxiety-related cognitive underperformance as explanations for the relation between math anxiety and underperformance in math.

### Establishing an index of in-the-moment anxiety about math

Given that we found an association between math anxiety and underperformance in math, we moved on to identifying measures of math-related state anxiety that could potentially explain this association. Prior work, however, has shown inconsistent correlations between traditional (trait) math anxiety and various measures of in-the-moment (‘state’) anxiety about math^49^. Additionally, there is debate about the utility of self-report vs physiological arousal as measures state anxiety^61–64^, and there is evidence that people tend to have fairly poor introspection into how much they are physiologically reacting to stressors^65^. Therefore, rather than deciding a priori on a single ‘best’ way to measure math-related state anxiety (i.e., in-the-moment anxiety associated with math), we instead measured multiple indices, including subjective self-report measures and several biological measures related to anxiety: heart rate (HR), skin conductance levels (SCL), and pre-ejection period (PEP). It is important to note here that we do not assume that these physiological measure *are* measures of anxiety, the psychological construct, as there are not one-to-one associations between psychological states and physiological measures^66^. Rather, these measures have been shown in previous work to be associated with anxious states (for a review, see Hodges^67^), and therefore may provide a useful—though indirect—on-line index of the degree of state anxiety a person is experiencing. In addition to collecting multiple physiological measures, we also aimed to clearly distinguish between physiological measures collected while anticipating vs during completion of a math task, as failure to do so may partly explain previous inconsistent findings in the literature. Taken together, the state anxiety indices we tested were self-reported state anxiety while anticipating math, physiological measures while anticipating math (HR, SCL, PEP), physiological measures while completing math (HR, SCL, PEP), and another self-reported state anxiety measure collected immediately following the completion of math. For a measure of state anxiety to explain math-anxious underperformance, that measure needs to be related to both math anxiety and math performance. The ‘best’ index of math-related state anxiety, for our current purposes, are therefore those that are significantly associated with both math anxiety and performance, and collecting this variety of measures allows us to take a data-driven approach to selecting which measure of anxiety to move forward with in our investigation.

We thus sought to identify which indices of math-related state anxiety, if any, correlated with both math anxiety and math performance. Importantly, testing several candidate measures meant testing a large number of correlations, which exposes one to the threat of Type I errors due to multiple comparisons. To account for this, we adjusted our alpha level (using the Bonferroni method correcting for 8 comparisons) from 0.05 to 0.003.

Furthermore, we sought to establish that an observed relation between a given math-related state anxiety measure and math anxiety/math performance was *specific to math*. To do so, when examining associations with math anxiety, we controlled for general anxiety, and when examining associations with math performance, we controlled for performance on the word task. In addition, when examining any associations involving a given math-related state anxiety index (e.g., PEP while completing math), we controlled for the corresponding index from the word blocks (e.g., PEP while completing the word task). Together, the above steps allowed for a data-driven approach to identifying the ‘best’ measure of in-the-moment anxiety about math, while also protecting against Type I errors and ensuring any effects are indeed specific to anxiety about math.

**Fig. 1:**
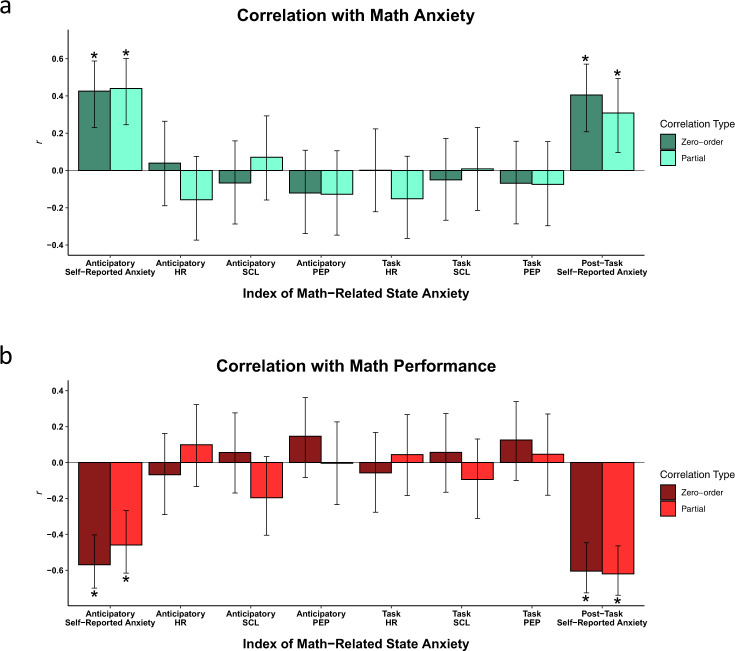
Associations between math-related state anxiety indices and math anxiety and performance. (**a**, **b**) show the extent to which self-reported and physiological indices of state anxiety from the Math blocks are associated with math anxiety and math performance, respectively. The darker colored bars indicate zero-order Pearson’s correlations. The lighter colored bars indicate partial correlations in which relevant covariates at both the level of the IV and DV are controlled for. Each partial correlation in (**a**) controls for general anxiety, and each partial correlation in (**b**) controls for word task performance. In both figures, each partial correlation also controls for the corresponding measure from the Word block (e.g., lighter bars showing the association between Anticipatory HR during the Math blocks and an outcome are controlling for Anticipatory HR during the Word blocks). Error bars represent 95% confidence intervals. * Indicates that the correlation coefficient is significant at the Bonferroni-adjusted alpha of *p* < 0.003.

Results shown in Fig. 1 indicate that of all the indices of state anxiety we tested, only participants’ subjective feelings of in-the-moment anxiety associated with math were related to both math anxiety and math performance. This was true for both the anticipatory self-reported state anxiety measure (association with math anxiety: *r*_partial_(79) = 0.440, *p* = 4E−5; association with math performance: *r*_partial_(79) = −0.459, *p* = 2E−5) and the post-task self-reported state anxiety measure (association with math anxiety: *r*_partial_(79) = 0.308, *p* = 0.005; association with math performance: *r*_partial_(79) = −0.620, *p* = 7E−10). These results are in line with past work by Conlon et al.^47^ that found that self-reported state anxiety measured before, during, and after an arithmetic fluency task are all significantly associated with both trait-level math anxiety and with math performance. Broadly consistent with past work (for a review, see Avancini & Szűcs^49^), none of the biological indices of state anxiety (HR, SCL, PEP) were significantly associated with either math anxiety or math performance (all *p*s > 0.05).

Together, these results suggest that people’s subjective ratings of how anxious they are feeling in-the-moment while either anticipating or having just completed math are viable candidates to explain math-anxious underperformance, and the objective measures of physiological arousal we collected are not. While both the anticipatory and post-task self-reported anxiety measures fulfill our criteria, in the rest of the results we focus on the anticipatory measure. This is because (A) multiple math anxiety interventions, like expressive writing and reappraisal^30,31^, aim to reduce anticipatory anxiety, so understanding the role played by anticipatory anxiety is most relevant to inform future intervention work; (B) if we focused on post-task anxiety, this would come with the risk that part of why the participant is anxious is simply because they performed poorly. Throughout the rest of the results, we therefore use anticipatory self-reported anxiety during the math blocks as our measure of ‘math-related state anxiety’ and refer to it with this label for the sake of simplicity. While past work has found differences between anxiety measured before, during, or after a test^68^, note that none of the inferences we draw below change if instead the post-task self-reported anxiety measures are used.

### Does in-the-moment anxiety explain math-anxious underperformance?

**Fig. 2:**
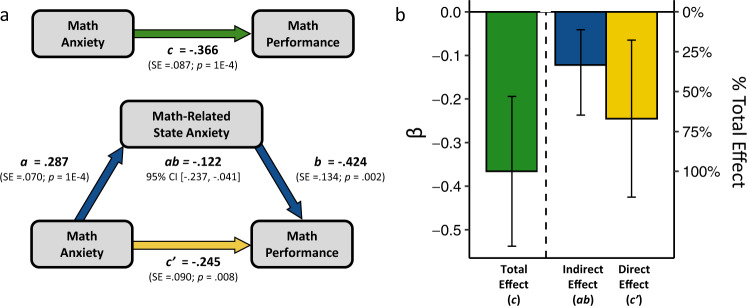
Mediating effect of math-related state anxiety on the math anxiety-performance link. (**a**) shows a mediation model that assesses the extent to which math-related state anxiety mediates the association between math anxiety and math performance. General anxiety, word task performance, and word task-related anxiety were controlled for. (**b**) visualizes the magnitudes of the total effect of math anxiety on math performance, the indirect effect of math anxiety on math performance through math-related state anxiety (i.e., the extent to which math-related state anxiety explains the math anxiety-performance association), and the direct effect of math anxiety on math performance independent of math-related state anxiety (i.e., the extent to which the math anxiety-performance association cannot be explained by math-related state anxiety). Error bars represent 95% confidence intervals.

To test the extent to which in-the-moment anxiety can explain why math-anxious people underperform in math, we ran a mediation analysis (Fig. 2) using the bootstrapping method (with 10,000 iterations) to generate standard errors and confidence intervals^69–71^. Below, all 95% confidence intervals we show are bootstrapped confidence intervals. Note that for all mediation analyses, we standardized all variables for ease of interpretation. To ensure effects were specific to math, we controlled for general anxiety, word-related state anxiety, and word task performance as general covariates in the mediation model.

Results in Fig. 2 demonstrate that math-related state anxiety does, indeed, explain (mediate) a significant portion of the link between math anxiety and math performance (Bootstrapped Indirect Effect = −0.122, 95% CI [−0.237, −0.041]), accounting for about a third (33.2%, 95% CI [11.2%, 64.6%]) of the association. However, this also implies that roughly two-thirds (66.8%) of the link between math anxiety and math underperformance was *not* explained by in-the-moment anxiety about math. (Indeed, the direct path remained statistically significant: *c*’ = −0.245, *p* = 0.008). On the one hand, consistent with the dominant view in the literature, these results provide the first direct support that heightened in-the-moment anxiety explains a significant portion of the relation between math anxiety and math underperformance. On the other hand, in-the-moment anxiety explains a much smaller portion of the relation than one would expect if this were the primary mechanism explaining math-anxiety underperformance in the majority of math-anxious individuals. In the next sections, we thus turn to the hypothesis that individual differences in emotion regulation capacity may help clarify whether and for whom in-the-moment anxiety, on its own, is sufficient to explain why math anxious individuals underperform in math.

### Does the link between math anxiety and in-the-moment anxiety (when faced with math) depend on differences in emotion regulation?

The above results provide evidence that in-the-moment anxiety when faced with math can explain a significant portion of the link between math anxiety and math underperformance. In the Introduction, we predicted that the emotion regulation skills a math-anxious person brings to the table may, in part, determine how anxious they feel when faced with math. Here we test this hypothesis.

We tested both subjective (self-report) and objective (physiological) indices related to emotion regulation commonly used in past research. In the online session, participants self-reported their tendency to attempt to downregulate negative emotions either by trying not to think about or express them—referred to as “suppression”—or by trying to think about their situation in a new way—referred to as “reappraisal”. Previous work on these self-report measures suggests that suppression is typically counterproductive for downregulating negative emotions while reappraisal is typically helpful^43^. Here we thus evaluate self-reported suppression and reappraisal separately (and refer to these variables as “ERQ-suppression” and “ERQ-reappraisal” to reflect the questionnaire they came from, the Emotion Regulation Questionnaire). Our physiological index related to emotion regulation was high-frequency heart rate variability (HF-HRV), which is often used as an indirect marker of neural self-regulation, emotion regulation ability, and how well individuals cope with stress^54–60^. Note that, as is the case for the physiological indices of state anxiety here, HF-HRV should not be thought of as a direct measure of emotion regulation, but rather as biological measure that has been associated with successful emotion regulation outcomes in past work. Further, because the majority of work supporting HF-HRV as a trait-level index of the efficacy of neural emotion regulation processes has relied on measures of resting baseline HF-HRV^54–56^, we follow that convention here. While state-level analyses of HF-HRV may be of interest to future work, the current design is not well-suited for such analyses because our measures of HF-HRV collected in anticipation of math and during the math task itself are collected after the self-reported state anxiety measure that it is meant to moderate, and thus we cannot be certain that self-reported state anxiety levels would not have an influence on the state-level HF-HRV metrics we obtain here. Note, though, that the inferences below do not substantially change if instead HF-HRV collected in anticipation of the math task or during the math task is used.

In sum, we tested whether the relation between math anxiety and in-the-moment anxiety about math depended on individual differences in indices of emotion regulation. We tested three measures of the latter: ERQ suppression, ERQ reappraisal, and baseline HF-HRV. Because we tested multiple measures, we adjusted our alpha (correcting for three comparisons) to α = 0.017. Because previous analyses (Fig. 1) showed that only self-reported in-the-moment anxiety ratings correlated with math anxiety, we focus on that measure here as well. Finally, general anxiety and in-the-moment anxiety ratings during the word blocks were used as covariates.

**Fig. 3:**
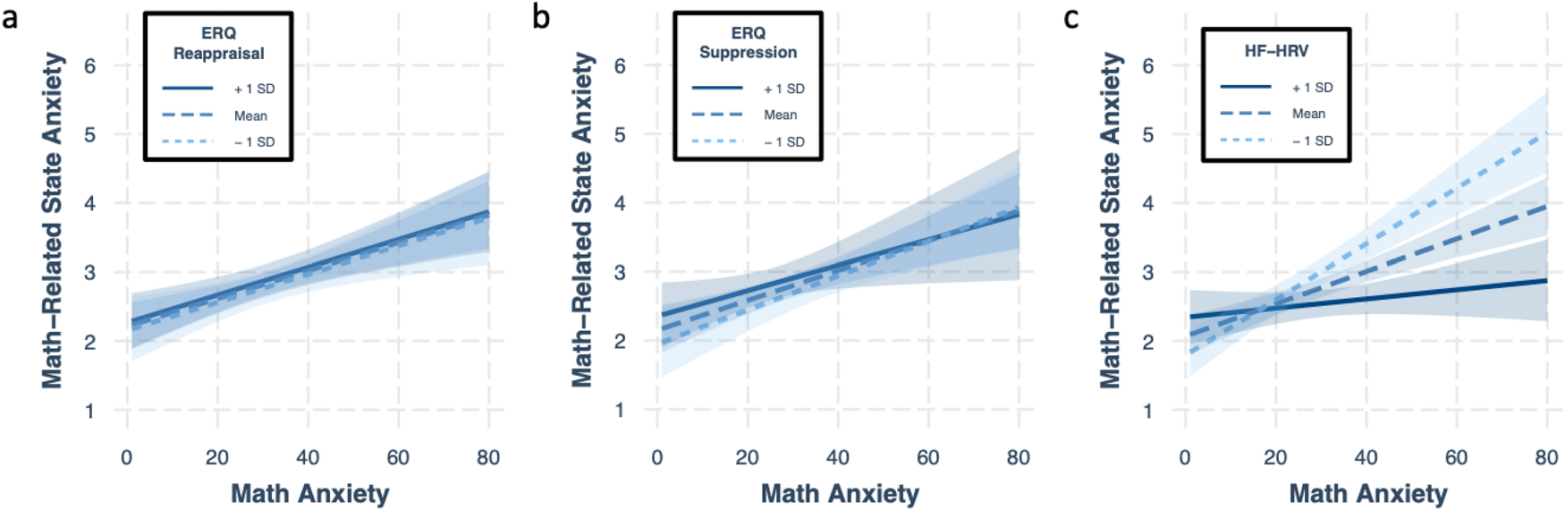
Moderating effect of emotion regulation indices on the math anxiety-state anxiety link. Results of simple slope analyses generated by multiple regressions that assess whether measures related to emotion regulation moderate the association between math anxiety and math-related state anxiety. In each multiple regression model, general anxiety and self-reported anxiety during Word blocks were included as covariates. The shaded area around each line of best fit reflects 95% confidence intervals.

Results visualized in Fig. 3 show that the link between math anxiety and in-the-moment anxiety did not significantly depend on either of the self-report measures of emotion regulation (*p*s > .05), but it did depend on HF-HRV (β = −0.259, *t*(73) = −4.59, *p* = 2E−5). Simple slopes shown in Fig. 3c help unpack this latter result. Namely, for those with low levels of HF-HRV (–1 SD), the strength of the relationship between math anxiety and math-related state anxiety was exacerbated (β = 0.621, *p* = 8E−11) relative to those with average HF-HRV levels (β = 0.362, *p* = 2E−7); for those with high levels of HF-HRV ( + 1 SD), the effect of math anxiety on math-related state anxiety was no longer significant (β = 0.103, *p* = 0.244).

In sum, these results show that the standard prediction that high math anxiety leads to heightened in-the-moment anxiety when faced with math is most pertinent for those with low-HF-HRV levels (a physiological index often associated with successful emotion regulation). The other side of this coin, however, is that for those with high HF-HRV levels, *math anxiety ceases to significantly predict in-the-moment anxiety associated with math altogether*. Given that results thus far have shown that math-related state anxiety explains a substantial portion of math-anxious underperformance (Fig. 2) and that just how anxious math-anxious people feel when faced with math depends on HF-HRV levels (Fig. 3), we next turn to the question of whether HF-HRV also plays a role in determining the extent of math-anxious underperformance.

### Does the extent of math-anxious underperformance depend on HF-HRV?

We next tested whether the relation between math anxiety and math performance depended on HF-HRV levels. Note that here we focused just on HF-HRV as our emotion regulation index, and we controlled for general anxiety and word performance. In the previous section (Fig. 3), we saw that how anxious math-anxious people felt in-the-moment when faced with math was dependent on HF-HRV levels, where those with low HF-HRV felt particularly anxious when faced with math and those with high HF-HRV felt much less anxious. It thus seems reasonable to predict here that low levels of HF-HRV would exacerbate the negative relation between math anxiety and math performance while high levels of HF-HRV would attenuate it.

**Fig. 4:**
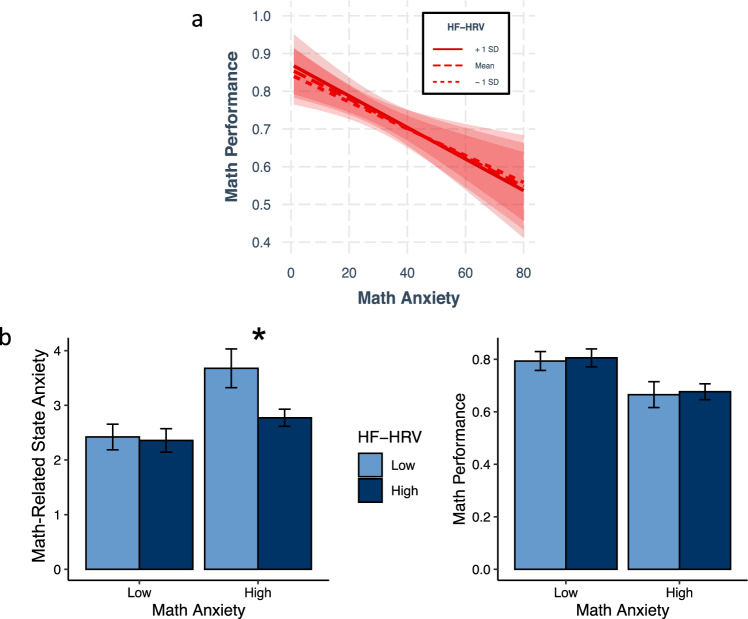
Unpacking the Moderating Role of HF-HRV. (**a**) shows that the magnitude of the association between math anxiety and math performance is not dependent on HF-HRV. (**b**) shows average levels of math-related state anxiety (left) and math performance (right) broken down by median splits on both math anxiety and HF-HRV. For the purposes of this figure, participants were considered “High” on math anxiety or HF-HRV if their score on that metric was above the median, and “Low” if it was at or below the median. Error bars reflect standard errors. * Indicates a significant difference as a function of HF-HRV at *p* < 0.05.

Perhaps surprisingly, this prediction was not supported by the data: the relation between math anxiety and math performance did *not* significantly depend on HF-HRV levels (β = −0.032, *t*(73) = −0.400, *p* = 0.691; trait anxiety and word performance were included in the model as covariates). For a visualization of this result, see Fig. 4a.

As this result may at first seem difficult to square with that in Fig. 3c, Fig. 4b uses a median-split of HF-HRV levels to visualize the results perhaps a bit more intuitively. From Fig. 4b (left), we see that in-the-moment anxiety is most elevated in those with high math anxiety and low HF-HRV. From Fig. 4b (right), we see that those high in math anxiety underperform in math (relative to those low in math anxiety), and the extent of this underperformance does not depend on HF-HRV levels. Note also that the timing of HF-HRV measurement did not impact the results of whether HF-HRV modulated math-anxious underperformance: while the measure of HF-HRV we focus on here is measured during baseline, HF-HRV measured during anticipation of math or during the math task similarly failed to moderate the link between math anxiety and math performance (Anticipatory HF-HRV: β = 0.052, *t*(70) = 0.557, *p* = 0.579; Task HF-HRV: β = 0.061, *t*(67) = −0.400, *p* = 0.515). Taken together, the above results suggest that while high HR-HRV can protect those who are math-anxious from feeling heightened in-the-moment anxiety when faced with math, this protection does not necessarily lead to better performance. One possibility these results raise is that different mechanistic pathways are needed to explain why math-anxious individuals underperform in math, and that the relevant mechanism depends the level of emotion regulation a person brings to the table (as indexed by HF-HRV). We turn to this possibility in the next section.

#### Testing for disparate mechanisms to explain math-anxious underperformance

From Fig. 2, we saw that while in-the-moment anxiety explains a significant proportion of math-anxious underperformance, it explained less (only about 33%) than expected based on the literature. When we examined individual differences in emotion regulation, for those *low* in HF-HRV, we found a pattern of results that strongly conformed to what one would expect if in-the-moment anxiety was the primary cause of math-anxious underperformance: heightened in-the-moment anxiety (Figs. 3c, 4b) and poor math performance (Fig. 4c). However, we also found that math-anxious people with *high* HF-HRV nevertheless underperformed in math to the same degree as those with low HF-HRV (Fig. 4c), despite feeling significantly less anxious (Figs. 3c, 4b). How can this be?

One explanation is that while the *magnitude* of the association between math anxiety and math performance may not depend on HF-HRV levels, the *mechanisms* by which this association is realized do depend on HF-HRV. In other words, math-anxious individuals who are high versus low in HF-HRV may underperform in math to the same degree, *but for different reasons*. To test this idea, we used conditional process analysis to ask whether the overall mediating effect of math-related state anxiety (shown above in Fig. 2) *depended on* HF-HRV levels^71^. To say this another way, we tested whether the extent to which math-related state anxiety explains math-anxious underperformance differs as a function of HF-HRV.

**Fig. 5:**
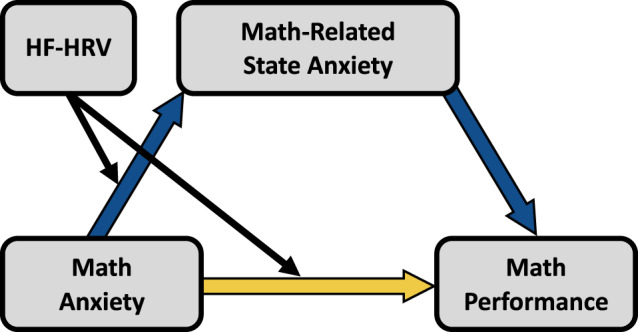
Conditional Process Model. Fig. 5 provides a conceptual version of the final conditional process model.

We began with a preliminary analysis using a full conditional process model that allowed for the possibility that HF-HRV moderated any of the paths (*a*, *b* and *c*’) in the mediation model shown in Fig. 2. (Covariates in the model were general anxiety, word task-related state anxiety, and word task performance.) Results from this model indicated significant moderation by HF-HRV of the link between math anxiety and math-related state anxiety (the *a* path; β = −0.255, *p* = 3E−5) and the direct effect between math anxiety and math performance after accounting for math-related state anxiety (the *c’* path; β = −0.270, *p* = 0.015), but not the link between math-related state anxiety and math performance (the *b* path; β = 0.142, *p* = 0.163). To preserve degrees of freedom and simplify interpretation, we therefore “pruned” the model by removing the moderation effect of the *b* path (consistent with best practices^71^). A conceptual summary of the final model is shown in Fig. 5.

**Fig. 6:**
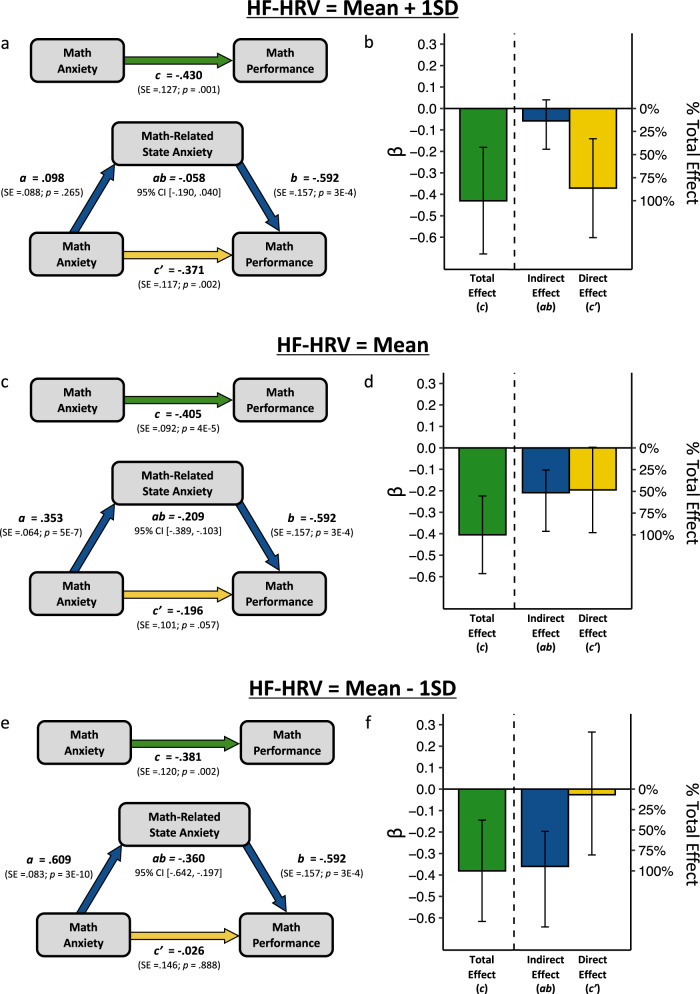
Mediating role of math-related state anxiety by HF-HRV levels. Figure shows the predicted mediation models for individuals at different levels of HF-HRV using simple slope analyses generated by the final conditional process model shown in Fig. 6. (In this model, the *a* and *c* paths were allowed to vary, while the b path was not because no significant moderation of the *b* path was observed; see text for details.) (**b**, **d**, **f**) visualize the magnitudes of the total effect of math anxiety on math performance (green), the indirect (mediation) effect via math-related state anxiety (blue), and the direct effect of math anxiety on math performance after controlling for math-related state anxiety (yellow). Error bars represent 95% confidence intervals for the beta estimates.

In the final model (Fig. 5), it is noteworthy that—despite holding the *b* path constant—HF-HRV significantly moderated not just the *a* path, but also the indirect effect (*ab* path: Index of moderated mediation = 0.151, 95% CI [0.077, 0.284]). This result is important as it indicates that the extent to which math-related state anxiety explains math-anxious underperformance indeed depends on HF-HRV levels. In particular, note that the moderated mediation effect is positive while the average indirect effect (Figs. 2, 6) is negative. This implies that as the value of the moderator increases (i.e., as HF-HRV increases), the indirect effect gets closer to 0 (less significant). In other words, for low levels of HF-HRV, the mediating (explanatory) effect of in-the-moment anxiety is relatively strong, and for high levels of HF-HRV, the mediating (explanatory) effect of in-the-moment anxiety is relatively weak. To make the implications of these findings clearer, Fig. 6 visualizes the mediation model at different levels of the moderator, HF-HRV, using a simple slopes approach. We further unpack these results below.

For those with average HF-HRV levels (Fig. 6c, d), math-related state anxiety explains about half of the link between math anxiety and math performance. This suggests that, for the average participant, math-related state anxiety plays a substantial role in explaining why math-anxious people underperform in math, but other factors are at play as well. (Note that estimates in this model in Fig. 6 differ slightly from those in Fig. 3 as a result of missing HF-HRV data from four participants; see Methods for details.)

For those with low-HF-HRV levels (–1 SD, Fig. 6e, f), the role played by in-the-moment feelings of anxiety is closely in line with the idea that in-the-moment anxiety is the chief cause of math-anxious underperformance. At low-HF-HRV levels, math anxiety is highly predictive of in-the-moment feelings of anxiety, and this in-the-moment anxiety in turn explains the vast majority of the link between math anxiety and math underperformance. One can see this in Fig. 6f where the blue bar (indirect effect) is nearly as high (94%) as the green bar (total effect), and the yellow bar (direct effect) is virtually at 0 (6%). More broadly, this result indicates that math-anxious people who are low in HF-HRV underperform *primarily* as a result of heightened feelings of in-the-moment anxiety.

For those with high HF-HRV levels (+1 SD, Figure a-b), the story is quite different. At high HF-HRV levels, math anxiety is not predictive of in-the-moment feelings of anxiety, and *the effect of math anxiety on math performance occurs almost entirely independently of math-related state anxiety*. One can see this in Fig. 6b where the *yellow* bar (direct effect) is nearly as high (86%) as the green bar (total effect), and the *blue* bar (indirect effect) is non-significant (14%). This result indicates that for those with high HF-HRV levels, feelings of in-the-moment anxiety are largely irrelevant for explaining why math-anxious people underperform in math. To be sure, highly math-anxious individuals even with high HF-HRV still underperform in math: the green bar (total effect) is still highly significant in Fig. 6b. Rather, it appears that something other than the dominant explanation in the literature—in-the-moment anxiety—is needed to explain this effect for those high in HF-HRV.

To summarize the moderated mediation results, we find that the prevalent idea that math-anxious underperformance in math is due to in-the-moment feelings of anxiety is only partially correct. More to the point, the extent to which explanation is accurate depends on a physiological index of emotion regulation: HF-HRV. For low levels of HR-HRV, the in-the-moment anxiety story appears to be correct; for high levels of HF-HRV, the in-the-moment anxiety story is largely incomplete; for those with average levels of HF-HRV, the answer seems to be somewhere in-between. More broadly, these results indicate that there is substantial heterogeneity in the mechanisms by which math anxiety and math performance are associated, and this heterogeneity occurs as a function of HF-HRV.

## Discussion

Math-anxious people consistently underperform in math. The most widely accepted explanation for *why* this underperformance occurs is that math-anxious people experience heightened anxiety when faced with math, and this in-the-moment anxiety interferes with performance. However, to our knowledge, this proposition has not been directly tested, relying instead on indirect evidence in which in-the-moment anxiety levels were not measured. Furthermore, despite the fact that emotion regulation processes are regularly the target of math anxiety interventions, the extent to which a priori differences in emotion regulation modulates the links between math-anxiety and in-the-moment anxiety, and between math-anxiety and math underperformance remains largely unknown. Thus, in the present study, we sought to systematically evaluate the extent to which the relation between math-anxiety and math underperformance can be explained by in-the-moment feelings of anxiety, and to assess whether the answer depends on individual differences in indices of emotion regulation.

We found that, consistent with what was predicted from the literature, in-the-moment feelings of anxiety before having to do math do indeed account for a significant portion math anxiety underperformance (Fig. 2a). However, contrary to this prediction, roughly two-thirds of the association between math anxiety and math performance remained unexplained by in-the-moment anxiety associated with math (Fig. 2b), suggesting that an account of math-anxious underperformance that relies on in-the-moment anxiety as the primary explanatory factor is incomplete. Examining individual differences in HF-HRV, a physiological index related to emotion regulation, provided a potential path toward a more complete explanation. The mediating effect of in-the-moment anxiety was nearly complete (94%, Fig. 6f) for individuals with low levels of HF-HRV (an indirect index of low neural emotion regulation circuit activity), but virtually absent for individuals with high levels of HF-HRV (14%, Fig. 6b). More broadly, our results provide evidence for heterogeneity (as a function of differences in HF-HRV) in the mechanisms by which math anxiety is linked to poor math performance. Perhaps of particular interest, for math-anxious individuals with high levels of HF-HRV, their feelings of in-the-moment anxiety cannot explain their underperformance in math, meaning that the link between high math anxiety and poor math performance in these individuals must be due to other mechanisms.

To our knowledge, our results provide the first direct support for the idea that feelings of in-the-moment anxiety are a significant reason why math-anxious people underperform in math. Perhaps just as important, they also provide clear evidence that, on average, in-the-moment anxiety only partially explains math-anxious underperformance. Moreover, the explanatory power of in-the-moment anxiety decreases as a biological index related to emotion regulation, HF-HRV, increases. This work strongly suggests that for the majority of math-anxious people, underperformance in math cannot be attributed solely to their feelings of anxiety actually faced with the prospect of doing mathematics.

Our findings raise two important questions about the mechanisms by which math anxiety is linked to math performance: A) What other mechanisms could explain math-anxious underperformance? B) Why would the relative importance of these other mechanisms depend on HF-HRV? One alternative mechanism to explain math-anxious underperformance that has been proposed in the literature is avoidance of math^5,15^. Here, the idea is that math-anxious individuals are more likely to avoid math when possible, and this can result in less practice over time and therefore less development of mathematical abilities. In general, we see this as a plausible alternative mechanism that should be investigated further, but in terms of the present results, it is unclear why the importance of math avoidance would depend on HF-HRV. For this to be the case, math avoidance would need to matter more for math-anxious people with higher HF-HRV (i.e., higher emotion regulation) and less for math-anxious people with lower HF-HRV; yet, past literature does not appear to give any indication this would be the case.

Another alternative mechanism that has been proposed to explain math-anxious underperformance is effort reduction: math-anxious people may simply expend less effort while doing tasks that involve math^72^. This, itself, could be a form of emotion regulation—if a math-anxious person cannot avoid doing math, one way to not experience heightened anxiety is to not engage fully with the task, possibly by distracting themselves by thinking about things that are not task-relevant^73^. One question, though, is why the relative importance of effort could plausibly differ as a function of HF-HRV. One of the proposals of Attentional Control Theory, an influential theory that aims to explain how anxiety impacts cognitive performance, is that anxiety and effort are positively correlated: to compensate for feelings of anxiety taking up cognitive resources, those who are anxious expend additional effort^2^. As a result, it could be that high levels of HF-HRV among math-anxious individuals are essentially exerting two effects when it is time to do math: reduced state anxiety levels (which we observed here) *and* reduced effort levels. Those lower in HF-HRV, in this account, would be expected to have higher anxiety levels but also higher effort levels. The relative tradeoff between anxiety and effort could explain why the degree of math-anxious underperformance is no different between those with high vs low HF-HRV.

It is important to note that the above explanations for why the mechanisms by which math-anxious underperformance is realized are dependent on HF-HRV levels are speculative. However, while the current work cannot directly address exactly what the alternative mechanisms that explain math-anxious underperformance are and why those mechanisms would differ as a function of HF-HRV, we are hopeful that this work will open up new lines of inquiry that will eventually allow us to understand the sources of heterogeneity in the mechanisms by which math anxiety is associated with underperformance in math. Including measures of previous math avoidance and in-task effort alongside the measures included in this work would enable the alternative mechanisms that may matter more for those high in HF-HRV to be tested directly in future work.

In addition, we believe these findings can be of more near-term practical utility as well. For math-anxious individuals with low-HF-HRV levels, in-the-moment anxiety appears to be perhaps the primary reason for their underperformance. As a result, interventions (like expressive writing^30^) that could prevent low-HF-HRV math-anxious people from experiencing heightened in-the-moment anxiety when faced with math should be expected to yield the highest benefits to math performance. On the other hand, emotion regulation interventions should be expected to be unlikely to reduce math-anxious underperformance of those with high HF-HRV, since their poor performance does not appear to be due to heightened in-the-moment anxiety (indeed, these individuals appear to avoid experiencing this heightened anxiety without any assistance). Optimally intervening on math-anxious underperformance for those with high HF-HRV, then, likely requires targeting different mechanisms. We believe the present results strongly suggest that measures of HF-HRV could predict which math-anxious individuals would most benefit from standard math anxiety interventions. While empirical work would need to demonstrate its efficacy directly, the present results suggest that measuring the HF-HRV of math-anxious people could allow for better targeting of math anxiety interventions, thereby increasing their overall efficacy and allowing for more optimal allocation of limited educational resources.

In addition to providing insights about math anxiety, the present findings also address broader questions about measurement of complex psychological constructs. Why is it that self-report indices of anxiety outperformed the physiological ones, but the opposite was true for indices of emotion regulation? We believe the explanation for this lies in the fact that (A) constructs like “anxiety” and “emotion regulation” are deeply multifaceted constructs, (B) in any given context, some of these facets are more likely to matter than others, and (C) different methods of attempting to measure these constructs emphasize different facets. “Anxiety”, for instance, is associated with particular bodily sensations, but also with a tendency to experience intrusive thoughts and worries^74^, and past work suggests that these intrusive thoughts may be more to blame for anxiety-induced performance deficits^75,76^. Through this lens, we believe that our self-report measure of in-the-moment anxiety may have explained the association between math anxiety and math performance while the physiological measures did not because the self-report anxiety measures tapped more into participants’ feelings of worry—the facet of anxiety potentially most relevant for performance—while the physiological measurements mostly reflected bodily sensations (which are only inconsistently associated with reports of worry^75^). This interpretation is also in line with appraisal-based accounts of math anxiety, which hold that individuals’ own interpretations of how they are feeling when faced with math are of central importance^14^.

Like anxiety, “emotion regulation” is also a multifaceted construct^77–79^. One distinction recently proposed is between emotion regulation *tendency*—what type of strategies people typically use to regulate emotions—and emotion regulation *capacity*—how effective people are able to be at regulating emotions^77^. The self-report Emotion Regulation Questionnaire^43^ is explicitly a measure of emotion regulation tendency, and gauges respondents’ proclivity to use particular emotion regulation strategies. On the other hand, HF-HRV, our physiological emotion regulation index, is seen as reflecting emotion regulation capacity—it is thought to indirectly reflect activity of key prefrontal-subcortical circuits that underlie effective emotion regulation^54–60^. In this context, emotion regulation *capacity* may be more relevant for allowing math-anxious individuals to avoid experiencing heightened anxiety when faced with math. We should note, however, that research is still unclear as to exactly how this regulation is accomplished. While it may seem surprising that HF-HRV, a physiological measure, is what modulated the association between math anxiety and self-reported in-the-moment anxiety, past work has shown that individuals with high HF-HRV report lower levels of worry^80,81^ and are better able to inhibit unwanted thoughts^82,83^. There is therefore substantial evidence that those with higher HF-HRV are able to effectively combat cognitive components of anxiety, likely owing to their stronger connections between prefrontal and subcortical regions.

While we believe the current work makes several advances in our understanding of why math-anxious people underperform, it is not without limitations. It is possible, for instance, that the extent to which in-the-moment anxiety can explain math-anxious underperformance is dependent on the particulars of the math task. Math-anxious underperformance on other forms of math (for instance, tests that require more prior knowledge) may be more or less explainable by in-the-moment anxiety. Additionally, while this work can speak to the mechanisms by which math anxiety is associated with math performance among a college-aged population, future work would be needed to assess whether the current findings would generalize to younger populations. These questions should be investigated further in future work. Moreover, future work could also measure visual ability (ensuring normal or corrected-to-normal vision) as well as histories of learning disabilities (e.g., dyslexia, dyscalculia) or ADHD to test whether these variables would affect the current results.

Through the present investigation, we made the following advances in our understanding of math anxiety: (1) We identified that self-report, but not physiological, indices of math-related state anxiety were associated with both math anxiety and math performance. (2) We found that in-the-moment feelings of anxiety when faced with math did, indeed, account for a substantial portion of the math anxiety-performance link, but a great deal of the association between math anxiety and math performance remained unexplained by math-related state anxiety. This strongly suggests that any account of math-anxious underperformance that focuses solely on in-the-moment anxiety is incomplete. (3) We showed that physiological (HF-HRV) but not self-report measures related to emotion regulation moderated the link between math anxiety and feelings of in-the-moment anxiety associated with math. Math-anxious people with high levels of HF-HRV were able to avoid experiencing heightened state anxiety when faced with math, and math-anxious people with low-HF-HRV levels experienced particularly heightened state anxiety. This suggests that having higher HF-HRV protects against feelings of in-the-moment anxiety among math-anxious individuals. (4) We further found that despite feeling less anxiety in-the-moment, math-anxious people with high HF-HRV levels nevertheless underperform in math to similar degrees as those with low-HF-HRV levels. The protective effects of high HF-HRV levels for state anxiety did not translate to protective effects for underperformance. (5) We therefore tested the idea that HF-HRV may moderate not the magnitude of the math anxiety-math performance link, but the mechanisms by which it is realized. Results suggest that for those lower in HF-HRV, math-related state anxiety explains the vast majority of math-anxious underperformance, but for those higher in HF-HRV, it explains almost none of why math-anxious people underperform. This finding provides evidence for heterogeneity (as a function of differences in HF-HRV) in the mechanisms by which math anxiety is linked to poor math performance.

In sum, we believe these findings have significant implications for both theory and practice. Theoretically, these findings suggest a need to seriously update our understanding of the reasons why math-anxious people underperform. In-the-moment anxiety appears to be a significant factor in explaining math-anxious underperformance, but its explanatory power depends on HF-HRV, and for most types of people (all but those lowest on HF-HRV), other factors are either as important or significantly more important. Moving forward, we believe the most complete understanding of math-anxious underperformance will come from considering multiple mechanisms together and from assessing whether the relative importance of these mechanisms differs as a function of other salient factors (like HF-HRV). Practically, the present work also may carry implications for interventions. Results strongly suggest that HF-HRV may be able to predict which students would benefit most from interventions that aim to decouple math anxiety and in-the-moment anxiety (those with low HF-HRV), which, if verified through empirical work, could allow for more effective targeting of limited educational resources. We believe that future work may need to take seriously the notion that multiple mechanisms can explain math-anxious underperformance, and that there is heterogeneity in the relative importance of these mechanisms across different math-anxious individuals. Doing so has the potential to give researchers and educators the most leverage to develop and deploy individualized interventions to optimally help every student who is struggling with math anxiety and allow them to fulfill their potential to succeed in mastering math.

## Methods

### Participants

Participation in the study took place in two parts, an online session and an in-lab session at Georgetown University. A total of 288 participants (198 female, mean age = 21.28; SD = 4.25) completed the online session, which involved completing surveys online for 30 min for course extra credit or a $5 Amazon gift card. After participants completed the online session, they were contacted via email with the opportunity to participate in the in-lab session. All participants who completed the online session were eligible to participate in the in-lab session, and participants were told that the in-lab session would involve completing cognitive tasks while undergoing psychophysiological recording (math was not specifically mentioned in the description of the in-lab session). The in-lab session always took place at least three days after a participant completed the online session and lasted 2 h. In-lab participants were compensated with course extra credit or at a rate of $10 per hour. All participants provided informed consent, and all procedures were approved by the Georgetown University Institutional Review Board. Of the 288 online participants, 83 (56 female; mean age = 22, SD = 5.63) completed the in-lab session. The analyses in the present work involve linking data collected during the online session with data collected during the in-lab session. As such, the N for the final analytic sample was 83 (though note that some physiology measures were missing for some participants; see the section *Missing Physiology Data and Inclusion Criteria* below for details).

### Online session

#### Procedure

In the online portion of the study, participants completed several surveys, many of which were collected for use in other studies and are not relevant to the present work. The order of all surveys was randomized across participants. Before beginning the survey battery, participants provided informed consent with an electronic signature, in line with the procedure approved by the Institutional Review Board. The survey measures that are relevant to the present study are described below.

##### Short math anxiety rating scale

Math anxiety was measured using the short math anxiety rating scale (sMARS^84^). Participants are presented 25 math-related situations (e.g., “Being given a set of addition problems to solve on paper”; “Getting ready to study for a math test”) and asked to indicate how anxious they would be in that situation from 0 (Not at all) to 4 (Very much). Scores range from 0 to 100, where higher scores reflect greater trait-level math anxiety. Note that math anxiety levels did not significantly differ between online participants who chose to participate in the in-lab session and those that did not (math anxiety among in-lab participants = 31.13, math anxiety among online-only participants = 33.37, *t*(286) = −0.94, *p* = 0.350). This indicates that those who self-selected into participating in the in-lab session did not differ meaningfully from those who did not in terms of trait-level math anxiety. Cronbach’s α for this measure was 0.954.

##### Trait Anxiety Inventory

General anxiety was measured using the trait component of the State-Trait Anxiety Inventory (STAI^85^). Participants respond to statements like “I feel nervous and restless” and “I am ‘calm, cool, and collected’” (reverse scored) on a scale from 1 (Almost never) to 4 (Almost always) to indicate how they generally feel. The scale contains a total of 20 items, and possible scores range from 20 to 80, where higher scores indicate greater general anxiety. This measure of general was included as a covariate to control for trait-level anxiety that is not specific to math. Cronbach’s α for this measure was 0.898.

##### Emotion regulation questionnaire

Self-reported tendencies to engage in two types of emotion regulation—reappraisal and suppression—were measured using the Emotion Regulation Questionnaire (ERQ^43^). In this scale, participants are shown 10 items and asked to indicate the extent to which they agree with each item on a scale from 1 (strongly disagree) to 7 (strongly agree). Of the 10 items, 6 are ‘reappraisal’ items, which ask participants to indicate how often they attempt to rethink or reframe the situation they are in to regulate their emotions (e.g., “I control my emotions by changing the way I think about the situation I’m in”; “When I’m faced with a stressful situation, I make myself think about it in a way that helps me stay calm”). The remaining 4 items are ‘suppression’ items, which ask participants to indicate how often they attempt to suppress emotions (e.g., “I control my emotions by not expressing them”, “When I am feeling negative emotions, I make sure not to express them”). Both reappraisal and suppression scores are calculated by averaging responses on the appropriate items, resulting in possible ranges from 1 to 7, where higher scores indicate greater tendencies to engage in that type of emotion regulation. Measures of reappraisal and suppression were collected as possible moderators of associations between math anxiety and indices of state-level anxiety and/or physiological reactivity associated with having to do math. Cronbach’s α was .848 for reappraisal and .753 for suppression.

### In-lab session

#### Procedure

After collecting written informed consent, the experimenter attached electrodes to the participant for electrocardiograph, impedance cardiograph, and skin conductance recording. Once these sensors were attached, participants completed an unrelated questionnaire (Need for Cognition^86^) to allow participants to adapt to having the sensors attached. Once participants finished this survey, a 3-min passive baseline measure of all psychophysiology measures was collected. After the baseline measurement, participants received instructions for and completed practice trials of all tasks. During the instructions, participants were told that the main portion of the in-lab session was divided into multiple blocks and that at the end of each block, they would complete either a math task or a word task. They were told that at the beginning of each block, they would be informed of whether the task at the end of the block would be the math task or the word task. They were also told that in the meantime, on some blocks, they would have to wait until the task began, and on other blocks, they would have to complete another cognitive task prior to starting the math or word task. Finally, they were told that they would occasionally be prompted to indicate their current anxiety level using the numbers on the keyboard.

The main portion of experiment consisted of eight blocks. At the beginning of each block, participants were given a cue (e.g., Upcoming Task: MATH) that indicated whether the task at the end of the block would be the math task or the word task. This cue appeared alone at the top center of the screen for 10 s and remained on the screen for the remainder of the block until either the math or word task began. Cues were always 100% veridical. Ten seconds after the cue first appeared, participants were asked to indicate their current anxiety level by selecting a number from 1 to 7 on the keyboard. The anxiety prompt remained on the screen for 10 s. After the anxiety prompt left the screen, a 3-min intervening period began between the anxiety response and the math or word task. During this intervening period, participants either looked at a fixation cross or completed one of three cognitive tasks (described below). After the 3-min intervening period, participants completed the cued task—math or word. Both the math and word tasks lasted for 3 min, immediately after which participants again indicated their anxiety level on a scale from 1 to 7. This concluded the block, which was followed by a 3-min rest period prior to the start of the next block. Four of the blocks concluded with a math task (and hence started with a math cue), and the other four blocks concluded with a word task (and hence started with a word cue). Within a block type (math or word) the intervening task—fixation or one of the three cognitive tasks—did not repeat. The order of blocks was randomized.

#### Tasks

##### Math and word tasks

The math and word tasks were adapted from Lyons and Beilock^45,46^. In the math task, participants were shown an equation on the screen (for example, “(7 × 5)–19 = 16”) and asked to indicate with a button press whether the equation was solved correctly or not (in this example, the correct response is “Yes”, because the equation is valid). All trials were of the form (a*b) – *c* = *d*, where *a* $\ne$ *b*, 5≤ *a* ≤ 9, 5≤ *b* ≤ 9, a*b ≥ 30, 15 ≤ *c* ≤19, and *d* > 0. Moreover, subtracting *c* from a*b always involved a borrow operation (e.g., “borrowing” from the tens place when subtracting 5 from 32). Half of the trials were valid (correct = ‘Yes’) and half were invalid (correct = ‘No’). For all invalid trials, *d* was ± 2 of the correct number. These trials corresponded to the ‘hard’ math problems from Lyons and Beilock^45,46^.

In the word task, participants were shown a string of 7 letters on the screen (for example, “etipsed”) and asked to indicate by button press whether or not, if the string was reversed, it would be a correctly spelled English word (in this example, the correct response is “Yes”, because, when reversed, that string spells “despite”). Half of the trials were valid (correct = ‘Yes’) and half were invalid (correct = ‘No’). All invalid trials comprised an actual word with the position of two of the internal letters flipped (for example, “noitenm” is the reverse of the word “mention” but with the n and e out of order, so when it is reversed, it spells “mnetion”). These trials corresponded to the ‘hard’ word problems from Lyons and Beilock^45,46^.

For both tasks, the problem appeared on the screen for a total of 5.5 s, followed by a 1.5 s inter-trial interval. Each task included 26 trials and lasted for 182 s. The screen did not change or advance when participants made their response—this decision was made to ensure a fixed total task duration to avoid biasing physiological recordings.

During practice (before the start of the 8 experimental blocks), participants completed 6 math trials and 6 word trials. Total math performance and word performance scores were computed as the accuracy participants achieved across all four blocks of each task, for a total of 104 trials of each task, with a possible range from 0 to 1.

##### Intervening cognitive tasks

The intervening cognitive tasks that occurred between the math or word cue and the math or word task were included to address research questions that are beyond the scope of the present work. The three intervening tasks were standard versions of the local-global task^87^, the antisaccade task^88^, and a free recall task^89^. Importantly, it should be noted that the results presented in this manuscript do not appreciably change if only the blocks that included no intervening task are included in analysis. In other words, the presence or absence of an intervening task did not impact any of the inferences drawn in this work; hence, in order to maximize statistical power (and thus accuracy of effect-size estimates), here we include data from all 8 blocks (4 math, 4 word).

#### In-lab session self-reported and psychophysiological measures

##### Self-reported anxiety ratings

At specific points throughout the experiment, participants received a prompt on the screen to indicate their current anxiety level via the keyboard. The prompt that appeared on the screen was “Please rate your anxiety level:” followed by a Likert scale from 1 to 7. In the instructions, participants were instructed to respond based on how they were currently feeling, and they were told that a response of 1 meant “Not at all anxious” and that a response of 7 meant “Extremely anxious”. Self-reported anxiety ratings were collected just after receiving the cue indicating what the upcoming task would be, the math task or the word task. Participants therefore provided 4 anxiety responses while anticipating the math task and 4 responses while anticipating the word task. These responses were averaged to produce measures of self-reported state anxiety for the math and word blocks, respectively. The reliability of both measures was high: Cronbach’s α = 0.90 for self-reported state anxiety for math blocks; Cronbach’s α = 0.84 for self-reported state anxiety for word blocks. We also collected the same self-reported state anxiety measures just after participants finished each math and word task.

##### Psychophysiological measures

During the in-lab portion of the study, several psychophysiological measures were collected. We collected measures of heart rate (HR) and skin conductance level (SCL). In addition, we also collected measures of pre-ejection period (PEP) and high-frequency heart rate variability (HF-HRV), which provide cardiac-based indices of sympathetic nervous system activity^51,90,91^ and parasympathetic nervous system activity^53,92,93^ respectively. Details of how these psychophysiological signals were measured can be found below.

These psychophysiological measures were collected continuously throughout the in-lab portion of the study. Key 3-min periods of these signals were analyzed for each measure. First, baseline measures of each signal were collected at the beginning of the in-lab portion of the study, in which participants were instructed to look at a fixation cross for 3-min while psychophysiological measures were collected. Another key 3-min period occurred in each block during the intervening period—the time between when participants were informed whether the upcoming main task would be the math or word task and when the main task actually began. During this time, participants either completed one of three intervening tasks or were instructed to focus on a fixation cross at the center of the screen. We refer to psychophysiological measures taken during this period as “Anticipatory” measures. Finally, all psychophysiological measures were also collected during the 3-min period in which participants completed the main task, either the math or word task. We refer to measures taken during this time as “Task” measures.

#### Cardiac psychophysiology measures

A standard lead II configuration was used to obtain the electrocardiogram (ECG). A BioNex two-slot mainframe (Mindware Technology, Gahanna, OH) connected to a personal computer was used for data collection. The ECG signal had a sampling rate of 1000 Hz and was analyzed using Mindware Technology’s HRV Analysis Software, Version 3.2.3. Artifacts and ectopic beats were removed via visual inspection and manual editing of the data, consistent with best practices^53^. While inspecting and editing the ECG signal, researchers were blind to the identity of the participant and to the experimental condition during which the signal was collected.

Pre-ejection period (PEP) and high-frequency heart rate variability (HF-HRV) were used as measures of sympathetic and parasympathetic cardiac control, respectively. PEP is calculated using impedance cardiography (ICG), and reflects the time period between ventricular depolarization and the opening of the aortic valve, which is largely under control of the sympathetic nervous system^50,51^. The ICG was obtained using the standard tetrapolar electrode system^51^. PEP measurements are in milliseconds, and a higher PEP value corresponds to lower levels of sympathetic cardiac control.

HF-HRV is the variability in heart rate that occurs in the respiratory frequency band (0.12–0.40 Hz), which has been shown to be relatively purely under parasympathetic control^53^. HF-HRV was derived using spectral analysis according to methods described in Berntson et al.^53^. The interbeat interval series was sampled at 4 Hz, resulting in an equal interval time series. The time series was then submitted to a fast Fourier transform. HF-HRV spectral power was integrated over the respiratory frequency band (0.12—0.40 Hz). The measure of HF-HRV is measured as the natural log of the heart period variance in the respiratory band, and is measured in ms^2^. Higher values of HF-HRV reflect higher levels of parasympathetic cardiac control.

Both PEP and HF-HRV were collected at several periods throughout the study—during the baseline recording, during the anticipatory period while participants await the upcoming task, during the task itself, and during the recovery period post-task. Each of these periods was 3 min in duration. Data were preprocessed in 1-min segments and averaged to produce the final analytic PEP and HF-HRV values for each time period. Note that because both the math and word tasks lasted for 182 s, the final 2 s of each task was not included in analysis. For the purposes of the current work, our primary interest in HF-HRV was as a trait-level index of emotion regulation. As a result, following the conventions of past work^54,56^, we focus on baseline HF-HRV in our analyses.

#### Electrodermal psychophysiology measure

Skin conductance levels (SCL^94^) were also measured at a sampling rate of 500 Hz using two electrodermal electrodes placed on the non-dominant hand. Electrodermal activity was analyzed using Mindware EDA Analysis software version 3.2.4. SCL is measured in micro Siemens, and higher values indicate greater electrodermal activity. To provide SCL values for specific time periods (e.g., “Anticipatory SCL” during math blocks), SCL over the relevant 3-min period was averaged.

#### Missing psychophysiology data and inclusion criteria

Due to user error with the Bionex, cardiac psychophysiological data was not collected for two participants. Additionally, due to an error with the connection between the stimulus computer and the computer connected to the Bionex, synchronized timing between the stimulus computer and the Bionex computer was lost for three participants. Finally, specific 1-min segments of psychophysiological data were removed from analysis due to artifacts caused by excessive motion. For baseline measures, at least two out of three 1-min segments were required to be present for a baseline measurement for that participant’s baseline data to be included in the final analytic dataset. Anticipatory and Task measures, like “Anticipatory PEP” and “Task PEP”, were made up of a total of 12 one-min segments (three each from a total of four blocks). For each block, at least two out of three 1-min segments were required to be present for that block’s measurement to be included in the overall measure, and all four blocks were required for that participant’s data to be included in the final analytic dataset.

If a participant was missing data for a given psychophysiology measurement (for instance, Anticipatory SCL), they were not included in analyses that made use of that measurement. Table 1 in the Results section shows the total number of participants with valid data for each measure.

### Reporting summary

Further information on research design is available in the Nature Research Reporting Summary linked to this article.

## Supplementary information

Supplemental Material

Reporting Summary

## Data Availability

The data supporting this work can be found on the Open Science Framework at the following link: https://osf.io/jru7k/?view_only=b91cdbc124994418a661cae13ab7f3db.
