# Supplementary material for: Does anxiety explain why math-anxious people underperform in math?
Source: NPJ Sci Learn. 2023 Mar 21;8:6. doi: 10.1038/s41539-023-00156-z (PMC10030629; doi:10.1038/s41539-023-00156-z)
Supplement: Supplementary file 1 — Supplemental Material [file 41539_2023_156_MOESM1_ESM.pdf]

Supplementary Table 1

	Math Anxiety	General Trait Anxiety	ERQ Reappraisal	ERQ Suppression	Math Performance	Word Performance	Anticipatory Self-Reported State Anxiety - Math	Anticipatory Self-Reported State Anxiety - Word	Post-Task Self-Reported State Anxiety - Math	Post-Task Self-Reported State Anxiety - Word	Task SCL - Math	Task SCL - Word	Task HR - Math	Task HR - Word	Task PEP - Math	Task PEP - Word	Task HF-HRV - Math	Task HF-HRV - Word	Anticipatory SCL - Math	Anticipatory SCL - Word	Anticipatory HR - Math	Anticipatory HR - Word	Anticipatory PEP - Math	Anticipatory PEP - Word	Anticipatory HF-HRV - Math	Anticipatory HF-HRV - Word	Baseline SCL	Baseline HR	Baseline PEP
General Trait Anxiety	.29																												
ERQ Reappraisal	-.05	-.27																											
ERQ Suppression	-.27	.20	.15																										
Math Performance	-.47	-.14	-.18	-.08																									
Word Performance	-.22	-.15	-.13	-.24	.60																								
Anticipatory Self-Reported State Anxiety - Math	.43	.25	.08	.11	-.57	-.56																							
Anticipatory Self-Reported State Anxiety - Word	.13	.07	.13	.13	-.34	-.59	.73																						
Post-Task Self-Reported State Anxiety - Math	.41	.26	.07	.03	-.60	-.53	.87	.67																					
Post-Task Self-Reported State Anxiety - Word	.27	.24	.06	.18	-.41	-.70	.79	.83	.80																				
Task SCL - Math	-.05	-.02	-.19	.06	.06	.11	-.19	-.20	-.19	-.20																			
Task SCL - Word	-.07	-.06	-.18	.04	.06	.09	-.19	-.17	-.19	-.18	.97																		
Task HR - Math	.00	-.05	.05	-.03	-.06	.10	.09	-.05	.01	-.07	.09	.08																	
Task HR - Word	.04	-.07	.06	-.05	-.06	.12	.07	-.04	.01	-.07	.08	.09	.96																
Task PEP - Math	-.07	.00	-.13	-.18	.13	.31	-.17	-.20	-.07	-.27	.14	.11	-.16	-.13															
Task PEP - Word	-.05	-.01	-.15	-.20	.09	.28	-.12	-.18	-.04	-.24	.15	.13	-.18	-.16	.94														
Task HF-HRV - Math	.02	.02	-.15	.04	.07	.06	-.17	-.06	-.10	-.06	.15	.14	-.44	-.41	-.13	-.09													
Task HF-HRV - Word	.00	.04	-.17	.01	.04	.09	-.17	-.07	-.07	-.09	.18	.14	-.37	-.41	-.06	-.02	.93												
Anticipatory SCL - Math	-.07	-.04	-.20	.06	.06	.11	-.16	-.19	-.17	-.18	.99	.97	.14	.14	.13	.15	.12	.15											
Anticipatory SCL - Word	-.07	-.05	-.20	.05	.08	.08	-.18	-.15	-.18	-.16	.97	.99	.08	.08	.12	.13	.14	.14	.97										
Anticipatory HR - Math	.04	-.04	.05	.00	-.07	.08	.08	-.08	.00	-.10	.11	.10	.98	.95	-.18	-.21	-.41	-.36	.13	.09									
Anticipatory HR - Word	.04	-.07	.03	-.03	-.05	.14	.07	-.05	.00	-.08	.07	.07	.96	.99	-.18	-.19	-.39	-.38	.13	.06	.96								
Anticipatory PEP - Math	-.12	-.07	-.17	-.16	.15	.32	-.18	-.21	-.06	-.24	.16	.13	-.19	-.14	.90	.84	-.05	.01	.17	.14	-.21	-.18							
Anticipatory PEP - Word	-.06	-.04	-.16	-.16	.10	.25	-.10	-.13	.00	-.19	.15	.13	-.25	-.21	.91	.93	-.02	.03	.15	.13	-.27	-.24	.92						
Anticipatory HF-HRV - Math	.06	.02	-.15	.01	.03	-.01	-.14	.01	-.05	.02	.03	.04	-.49	-.44	-.09	-.04	.90	.82	.04	.05	-.50	-.44	-.03	.02					
Anticipatory HF-HRV - Word	.03	.07	-.17	-.01	.04	.03	-.13	-.04	-.04	-.01	.15	.12	-.42	-.44	.00	.00	.89	.93	.12	.12	-.42	-.44	-.01	.03	.85				
Baseline SCL	-.01	.00	-.22	.01	.09	.09	-.11	-.11	-.16	-.15	.78	.77	.12	.11	.12	.11	.16	.20	.81	.79	.12	.10	.10	.11	.15	.22			
Baseline HR	.10	-.01	.03	-.01	-.14	.00	.14	-.06	.07	-.01	-.03	-.05	.87	.86	-.24	-.23	-.33	-.30	.01	-.05	.89	.88	-.21	-.26	-.42	-.39	.07		
Baseline PEP	-.10	-.05	-.14	-.18	.09	.27	-.13	-.12	-.04	-.23	.04	.01	-.27	-.25	.83	.82	.02	.09	.04	.02	-.29	-.28	.83	.88	.05	.12	.04	-.28	
Baseline HF-HRV	.05	-.10	-.07	-.11	.05	.08	-.19	-.08	-.14	-.08	.02	.02	-.33	-.33	-.03	.01	.61	.57	.02	.02	-.37	-.31	-.04	.01	.75	.62	.14	-.44	.02

Supplementary Table 1: Zero-order correlations for all variables are shown. Correlations with a magnitude greater than .22 are significant at $p < .05$.
