# Supplementary material for: Does anxiety explain why math-anxious people underperform in math?
Source: NPJ Sci Learn. 2023 Mar 21;8:6. doi: 10.1038/s41539-023-00156-z (PMC10030629; doi:10.1038/s41539-023-00156-z)
Supplement: Supplementary file 2 — Reporting Summary [file 41539_2023_156_MOESM2_ESM.pdf]

nature portfolio

Corresponding author(s): Richard Daker

Last updated by author(s): Sep 28, 2022

Reporting Summary

Nature Portfolio wishes to improve the reproducibility of the work that we publish. This form provides structure for consistency and transparency in reporting. For further information on Nature Portfolio policies, see our Editorial Policies and the Editorial Policy Checklist.

Statistics

For all statistical analyses, confirm that the following items are present in the figure legend, table legend, main text, or Methods section.

n/a	Confirmed	
☐	☒	The exact sample size (*n*) for each experimental group/condition, given as a discrete number and unit of measurement
☐	☒	A statement on whether measurements were taken from distinct samples or whether the same sample was measured repeatedly
☐	☒	The statistical test(s) used AND whether they are one- or two-sided<br>*Only common tests should be described solely by name; describe more complex techniques in the Methods section.*
☐	☒	A description of all covariates tested
☐	☒	A description of any assumptions or corrections, such as tests of normality and adjustment for multiple comparisons
☐	☒	A full description of the statistical parameters including central tendency (e.g. means) or other basic estimates (e.g. regression coefficient) AND variation (e.g. standard deviation) or associated estimates of uncertainty (e.g. confidence intervals)
☐	☒	For null hypothesis testing, the test statistic (e.g. *F*, *t*, *r*) with confidence intervals, effect sizes, degrees of freedom and *P* value noted<br>*Give P values as exact values whenever suitable.*
☒	☐	For Bayesian analysis, information on the choice of priors and Markov chain Monte Carlo settings
☒	☐	For hierarchical and complex designs, identification of the appropriate level for tests and full reporting of outcomes
☐	☒	Estimates of effect sizes (e.g. Cohen's *d*, Pearson's *r*), indicating how they were calculated

Our web collection on statistics for biologists contains articles on many of the points above.

Software and code

Policy information about availability of computer code

Data collection	Qualtrics; E-Prime version 2
Data analysis	R

For manuscripts utilizing custom algorithms or software that are central to the research but not yet described in published literature, software must be made available to editors and reviewers. We strongly encourage code deposition in a community repository (e.g. GitHub). See the Nature Portfolio guidelines for submitting code & software for further information.

Data

Policy information about availability of data

All manuscripts must include a data availability statement. This statement should provide the following information, where applicable:

- Accession codes, unique identifiers, or web links for publicly available datasets
- A description of any restrictions on data availability
- For clinical datasets or third party data, please ensure that the statement adheres to our policy

The data supporting this work can be found on the Open Science Framework at the following link: https://osf.io/jru7k/?view_only=b91cdbc124994418a661cae13ab7f3db

Human research participants

Policy information about studies involving human research participants and Sex and Gender in Research.

Reporting on sex and gender	Data on gender has been included in the available data file. Gender was not a key focus of this paper, but we reran the key analyses and found that the same findings hold if gender is controlled for, suggesting that gender differences do not drive our results.
Population characteristics	See below
Recruitment	Convenience sample. Most participants are either psychology students who want extra credit or students who participated for cash. Recruitment practices are unlikely to have affected the associations between variables collected in this sample.
Ethics oversight	Georgetown University IRB

Note that full information on the approval of the study protocol must also be provided in the manuscript.

Field-specific reporting

Please select the one below that is the best fit for your research. If you are not sure, read the appropriate sections before making your selection.

☐ Life sciences ☒ Behavioural & social sciences ☐ Ecological, evolutionary & environmental sciences

For a reference copy of the document with all sections, see nature.com/documents/nr-reporting-summary-flat.pdf

Behavioural & social sciences study design

All studies must disclose on these points even when the disclosure is negative.

Study description	Quantitative
Research sample	Georgetown University undergraduates; 67% female, mean age =22. Findings generalize to university students, other work needed to test generalizability as we note in our Discussion.
Sampling strategy	Convenience sample; post-hoc power curve analyses informed by previous meta-analytic effect sizes indicated that the sample size was suitable to detect or reject a wide range of practically significant indirect effect sizes in mediation analyses
Data collection	Computer; only participant and experimenter in the room; experimenter blind to hypotheses
Timing	Start: January 2018, Stop: December 2018
Data exclusions	Data exclusions detailed in Methods section. Full or partial missing physiological recording data was the reason for all data exclusions.
Non-participation	No participants dropped out
Randomization	Not randomized

Reporting for specific materials, systems and methods

We require information from authors about some types of materials, experimental systems and methods used in many studies. Here, indicate whether each material, system or method listed is relevant to your study. If you are not sure if a list item applies to your research, read the appropriate section before selecting a response.

Materials & experimental systems

n/a	Involved in the study
☒	☐ Antibodies
☒	☐ Eukaryotic cell lines
☒	☐ Palaeontology and archaeology
☒	☐ Animals and other organisms
☒	☐ Clinical data
☒	☐ Dual use research of concern

Methods

n/a	Involved in the study
☒	☐ ChIP-seq
☒	☐ Flow cytometry
☒	☐ MRI-based neuroimaging
